# Quantitative evaluation of upper urinary tract pump function in pigs with acute unilateral lower ureteral obstruction by 640-slice dynamic volume CT

**DOI:** 10.1186/s12894-021-00887-4

**Published:** 2021-09-02

**Authors:** Chongwen Mao, Cong Peng, Song Li, Liling Chen, Mengjing You, Kewei Fang, Shutian Xiang, Yunshan Su

**Affiliations:** 1 Department of Radiology, The Fourth Affiliated Hospital of Kunming Medical University, #176 Qingnian Road, Kunming, 650021 Yunnan China; 2 Department of Radiology, The Central Hospital of Wuhan, The Tongji Medical College, Huazhong University of Science and Technology, Wuhan, Hubei China; 3 Department of Urology, The Fourth Affiliated Hospital of Kunming Medical University, Kunming, Yunnan China; 4 Department of Clinical Skills Center, The Kunming Medical University, Kunming, Yunnan China; 5 Department of Urology, The Second Affiliated Hospital of Kunming Medical University, Kunming, Yunnan China

**Keywords:** Urethral obstruction, Dynamic volumetric CT, Renal pelvis constant pressure perfusion test, Upper urinary tract urodynamics, Pump function

## Abstract

**Background:**

It is a challenging problem to differentiate obstructive hydronephrosis from noninvasive evaluation of renal pelvis and ureteral motility in patients. The purpose of this study was to explore the value of 640-slice dynamic volume CT (DVCT) in the quantitative measurement of upper urinary tract (UUT) pump function after acute unilateral lower ureteral obstruction in pigs.

**Methods:**

In this study, a perfusion pig model was constructed by constant pressure perfusion testing of the renal pelvis and left nephrostomy. The perfusion and pressure measuring devices were connected to create a state of no obstruction and acute obstruction of the lower part of the left ureter. After successful modelling, continuous dynamic volume scanning of the bilateral renal excretion phase was performed with 640-slice DVCT, and pump functions of the renal pelvis and part of the upper ureter were calculated and analysed. No obstruction or acute obstruction of the lower part of the left ureter was observed. Pump functions of the renal pelvis and part of the upper ureter were determined**.**

**Results:**

The results showed that after LUUT fistulostomy, the time difference between the average UUT volume and positive volume value increased gradually, and the calculated flow velocity decreased, which was significantly different from that of the RUUT. The volume difference of the LUUT increased significantly in mild obstruction. In the bilateral control, the volume change rate of the LUUT increased with mild obstruction and decreased with severe obstruction, and there was a significant difference between the left and right sides.

**Conclusion:**

The continuous dynamic volume scan and measurement of 640-slice DVCT can obtain five pump function datasets of UUT in pigs with acute lower ureteral obstruction.

## Background

It is a challenging problem to differentiate obstructive hydronephrosis during noninvasive evaluation of the renal pelvis and ureteral motility in patients with obstructive and nonobstructive hydronephrosis [1]. At present, the evaluation of urodynamics of the upper urinary tract in clinical work mainly depends on noninvasive imaging examinations, such as ultrasound, intravenous pyelography, CT, MRI [2], radionuclide and dynamic near infrared fluorescence imaging [3], and these imaging methods can only speculate regarding the functional changes of the upper urinary tract based on morphological changes, and only some semiquantitative data have been obtained [4, 5]. The clinical quantitative evaluation of upper urinary tract urodynamics still depends on classical upper urinary tract urodynamic tests, that is, the renal pelvis constant pressure perfusion test and renal pelvis constant flow perfusion pressure test (Whitaker test) [6]. However, because the above two tests are invasive tests, they are only used in patients with nephrostomy [7].

In this experiment, a pig model of renal pelvis constant pressure perfusion (CPP) was established, and the upper urinary tract under different conditions of lower ureteral obstruction was scanned by a 640-slice dynamic volume CT scanning technique. Later, data were calculated and analysed to obtain the change rule of pump function of the upper urinary tract under different obstruction conditions to determine an imaging method for the noninvasive quantitative evaluation of upper urinary tract urodynamics instead of CPP. It is expected that this approach can replace the renal pelvis CPP test as an effective method for noninvasive quantitative imaging evaluation of UUT urodynamics.

## Methods

### Animals

Experimental animal modelling and grouping of healthy Diannan small-eared pigs were provided by the Experimental Animal Department of Kunming Medical University (Experimental Animal Licence No.: SYXK (Yunnan)). Ten pigs weighing 25 kg, half male and half female, were raised by the Experimental Animal Center of Kunming Medical University. The feeding conditions were as follows: room temperature 21 °C, air humidity 50% to 60%, light changes naturally with day and night, and free access to water. The kidneys of 10 pigs were divided into the left urinary tract as the experimental group and the right urinary tract as the control group. The process of this experiment was in accordance with regulations (Ministry of Science and Technology of the People's Republic of China, guidance on being kind to Experimental Animals, 2006-09-30) and was approved by the Medical Ethics Committee of Kunming Medical University.

### Nephrostomy

Before anaesthesia, all experimental pigs were prevented from drinking and fasted for 12 h before the experiment, followed by intraperitoneal injection of 3% pentobarbital sodium at 10 mg/kg, establishment of an ear vein channel and maintenance of intravenous infusion after successful induction of anaesthesia, tracheal intubation and connection of the ECG monitor. An initial dose of 3% pentobarbital sodium 7.2 mg/kg (administration rate 1–2 ml/min) was given. 25–30% of the initial dose should be added at 30 min intervals according to the anesthetic condition of the animal (such as agitation and pain reaction). After successful anaesthesia, the pig was fixed in the right lying position, a left subcostal incision was made, the muscle layer and peritoneum were cut open with an electric knife, and the left kidney was exposed. Next, the left ureter on the anterior medial and inferior side was found and separated in front of the psoas major muscle, retaining the upper segment of the ureter for 3 to 4 cm, and keeping the ureter and renal hilum in a straight line, a catheter sheath was used to protect a 0.035 hard guide wire inserted retrograde into the renal pelvis from the ureteral incision and out of the kidney. The wound of the renal cortex was enlarged with the tip of the catheter sheath and then withdrawn. Then, the F8 fistulostomy tube with a length of 15 cm was inserted, the guide wire was pulled to send the fistulostomy tube into the renal pelvis, and the operator touched the tube head in the renal pelvis and fixed it. The F12 suction tube was placed on the control valve and inserted into the upper ureteral stump. The distal end of the tube was more than 3 cm from the ureteropelvic junction, the distal end was drawn out of the body and connected to the urine bag, and a catheter was inserted into the lower ureteral stump on the left to drain bladder urine. After fixing the position of the left nephrostomy tube and catheter, the kidney was  moved back to original positon, the bleeding was thoroughly stopped, the abdominal cavity was closed layer by layer with sutures, and the incision was closed with a film dressing.

### Perfusion

**Fig. 1:**
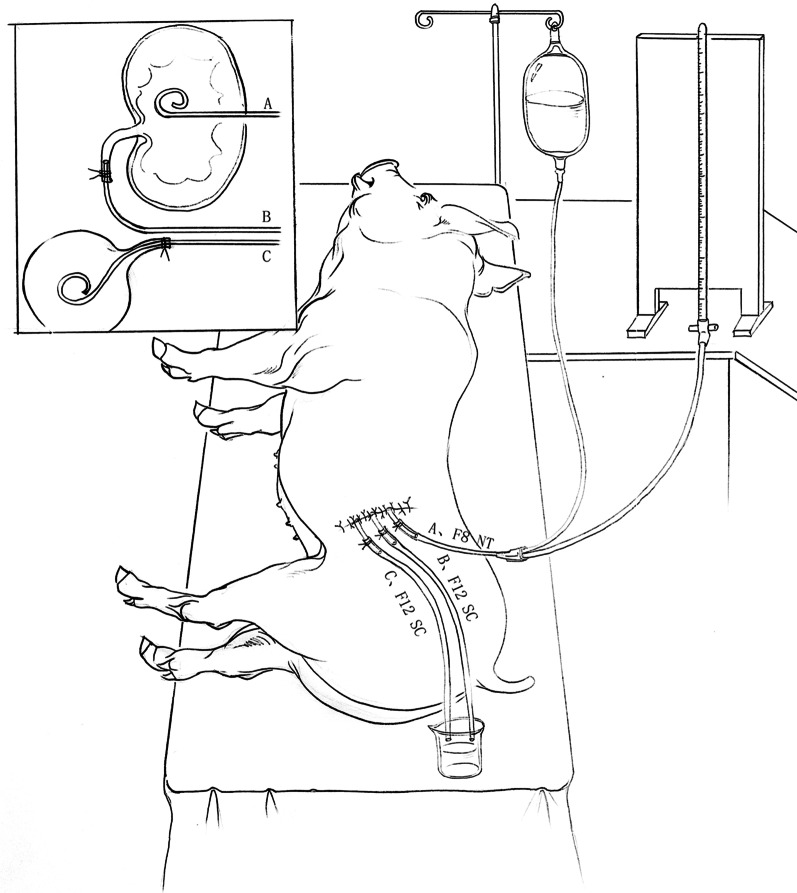
Model of animals receiving perfusion

Tees were established for the left kidney CPP test to connect the manometer tube, perfusion tube and fistulostomy tube. All tubes were fully vented, and imaging was performed to ensure no bubble interference. The perfusion tube and manometer tube were suspended on the infusion rack, and the liquid level height of the manometer tube and perfusion tube was 20 cm (20 cmH_2_O) above the renal pelvis plane (marked as 0 scale). The perfusion solution was a mixture of iodopropamide saline solution (10%), and a small amount of methylene blue was added for easy observation; the perfusion tube and the manometer tube were modified with the same type and length of pipeline (Fig. 1).

### Detection

The control valve of the drainage tube at the upper end of the left ureter was set to 100, 50 and 25% open at an interval of 30 min, and the ureter was brought into a non-obstruction, mild or severe obstruction state and scanned by CT. In the experiment, the height of the perfusion solution was adjusted to control the constant perfusion pressure. The amount of liquid flowing out of 1 cm was measured when the perfusion fluid height decreased. Then, each time the liquid was removed, the perfusion bottle was raised by 1 cm to maintain a constant pressure. After the beginning of constant pressure perfusion, the perfusion velocity underwent a process of first fast, then slow, then slightly faster and finally steady, which lasted for 5 to 10 min, so the flow velocity was recorded 10 min after the beginning of perfusion, and then CT scanning was performed. The left ureteral obstruction state was set to measure the flow rate under the constant pressure perfusion of the left renal pelvis, the control valve was set to 100% open, and when the flow rate was more than 10 ml/min (0.166 ml/s), it was in a non-obstructive state; the control valve was set to a 50% open state, and the mild obstruction state was measured when the flow rate was 4–10 ml/min (average approximately 0.116 ml/s). The control valve was set to 25% open, and the flow rate was less than 4 ml/min (0.067 ml/s) for severe obstruction [8]. The bilateral kidneys and upper ureter of pigs were scanned by volume enhancement when the flow velocity was stable in the state of no obstruction, mild obstruction and severe obstruction. Scanning methods: Continuous dynamic volume scanning was performed. The auricular vein was injected with 30 ml of nonionic contrast agent iopramine (370 mgI/ml) at a speed of 3.5 ml/s with a double-barrel high-pressure syringe (OptivantageHD), and then 20 ml of saline was injected at the same flow rate. The scanning program was triggered after entering the excretion period. Scanning parameters: tube voltage 80 kV, tube current 100 mAs, rotation time 0.5 s, collimation 0.5 mm × 320 mm, scan thickness 0.5 mm, and scan interval 2 s, for a total of 10 scans. The examination bed was not moved during scanning, and 10 consecutive groups of volume images with an interval of 2 s during excretion of the double kidneys and upper ureter were obtained. The radiation dose met the required limits (DLP = 473 mGy.cm; ED = 473*k = 7.095 mSv, k = 0.015 mSv/(mGy.cm)).

### Imaging

**Fig. 2:**
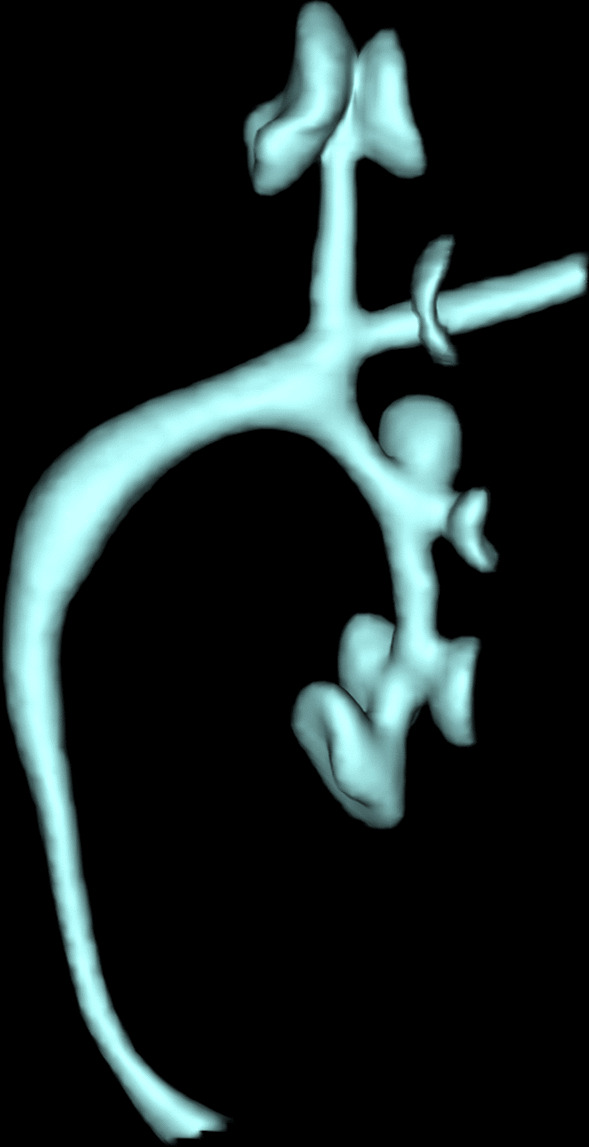
Volume data of the renal pelvis and upper ureter involved in CT detection

Image postprocessing and subjective evaluation of image quality: The original volume data of 10 groups of kidneys and upper ureters obtained by 640-slice dynamic volume CT scan were imported into the Vitreafx workstation (Vitreafx, Version 4.60) for postprocessing operation. The volume data of the renal pelvis and part of the upper ureter were obtained by setting the unified CT value of interest, as shown in Fig. 2. In the 10 groups, the development of the renal pelvis and upper ureter was good, part of the anatomical details were not clear, and there could be local artefacts, but they did not affect the volume calculation.

### Data processing

All images were analysed and measured under the guidance of two experienced radiologists. The pump function values of the left and right renal pelvis and upper ureter were measured: the average volume of contrast medium (ml), volume difference (ml), time difference (s), volume change rate (%) and urine flow rate (ml/s) were calculated in all renal pelvis and renal calyx images and part of the upper ureter (above the lower pole plane) in each scanning field 3 times.

The formula is as follows:

$${\text{Volume\,average}} = \frac{{{\text{Sum\,of\,continuous\,multi - phase\,volume\,values\,of\,ipsilateral\,urinary\,tract}}}}{{{\text{Number\,of\,periods}}}}\quad ({\text{ml}})$$

$$\begin{aligned} & {\text{Volume\,difference}} \\ & \quad = {\text{The\,maximum\,value\,of\,volume\,data\,obtained\,by\,multiple\,collection\,of\,ipsilateral}} \\ & \quad \quad {\text{urinary\,tract}} - {\text{Minimum\,value}}\quad ({\text{ml}}) \\ \end{aligned}$$

$$\begin{aligned} & {\text{Time\,difference}} \\ & \quad = {\text{The\,maximum\,time\,point\,in\,the\,volume\,data\,obtained\,by\,multiple\,collection\,of}} \\ & \quad \quad {\text{ipsilateral\,urinary\,tract - Minimum\,point\,in\,time}}({\text{s}}) \\ \end{aligned}$$

$${\text{Volume\,change\,rate}} = \frac{{{\text{Ipsilateral\,volume\,difference}}}}{{{\text{Average\,volume\,of\,ipsilateral\,side}}}} \times 100\%$$

$${\text{Calculate\,the\,flow\,rate\,of\,urine}} = \frac{{{\text{Ipsilateral\,volume\,difference}}}}{{{\text{Ipsilateral\,time\,difference}}}}\quad ({\text{ml/s}})$$

The average values measured 3 times by 2 physicians were recorded and calculated. At the same time, the urine flow velocity (ml/s) of drainage to the end of the urine bag was recorded when the left ureter formed nonobstruction and mild and severe obstruction.

### Statistical analysis

All data were analysed by SPSS 23.0 statistical software. The mean and standard deviation of all values were calculated on one side. The groups were divided into two groups according to the left and right side, a paired sample t test was used for the comparison of normal distribution, a paired rank sum test was used for the comparison of skewness distribution, and the difference was statistically significant (*P* < 0.05).

## Results

Among the 10 pigs, 2 pigs could not be studied because of the failure of left nephrostomy. The experimental results of 8 pigs were introduced in this paper.

The urine flow velocity data from the upper ureteral stump to the urine bag of 8 pigs were recorded, which showed that the urine flow velocity of the CCP test decreased linearly in normal and different obstructive states, which met the requirements of the CCP test.

**Fig. 3:**
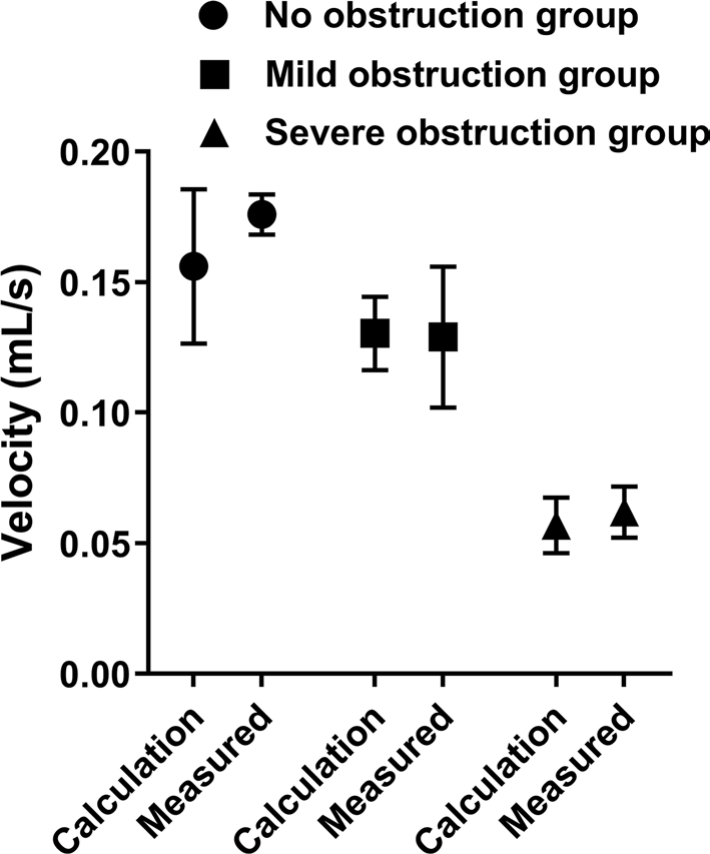
Comparison of left urinary tract calculation and measured velocity

**Fig. 4:**
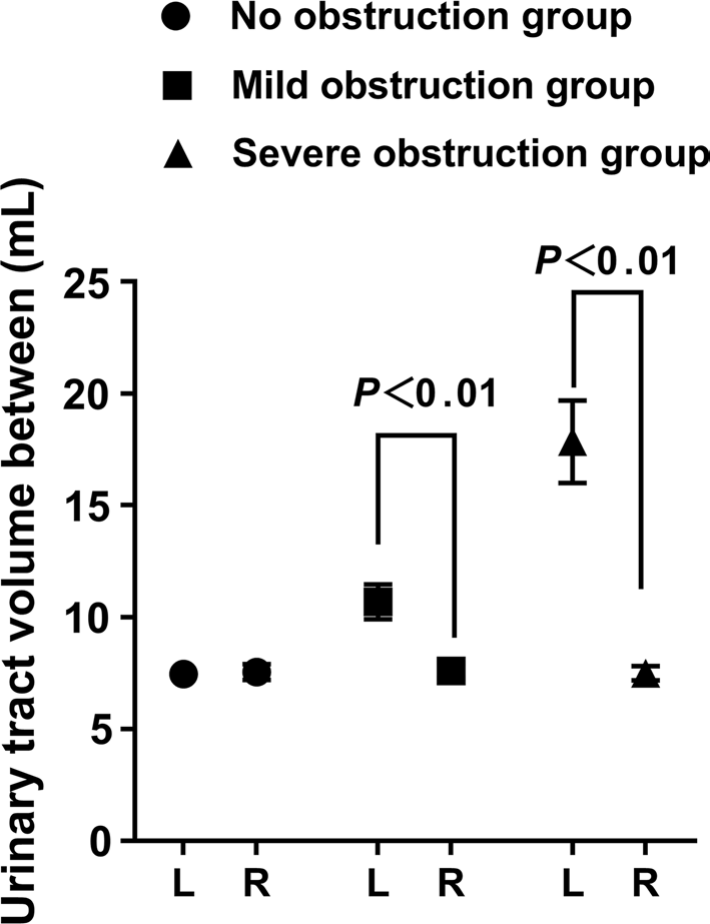
Comparison of mean upper urinary tract volume between different groups

**Fig. 5:**
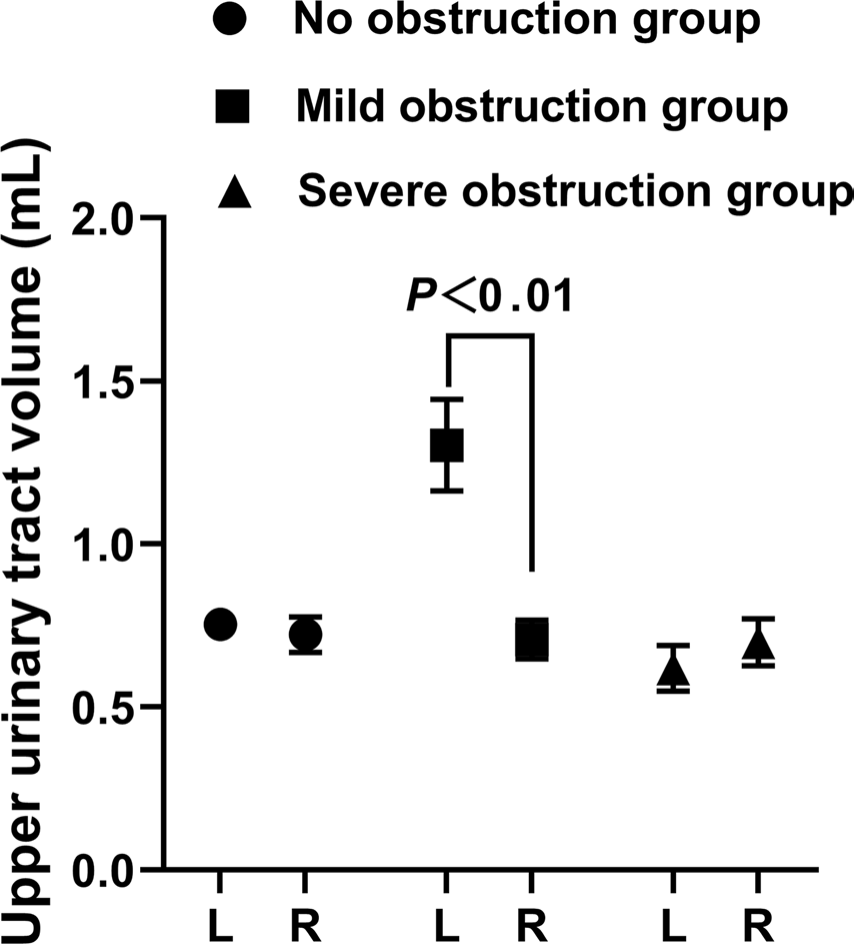
Comparison of extremum differences in upper urinary tract volume between different groups

**Fig. 6:**
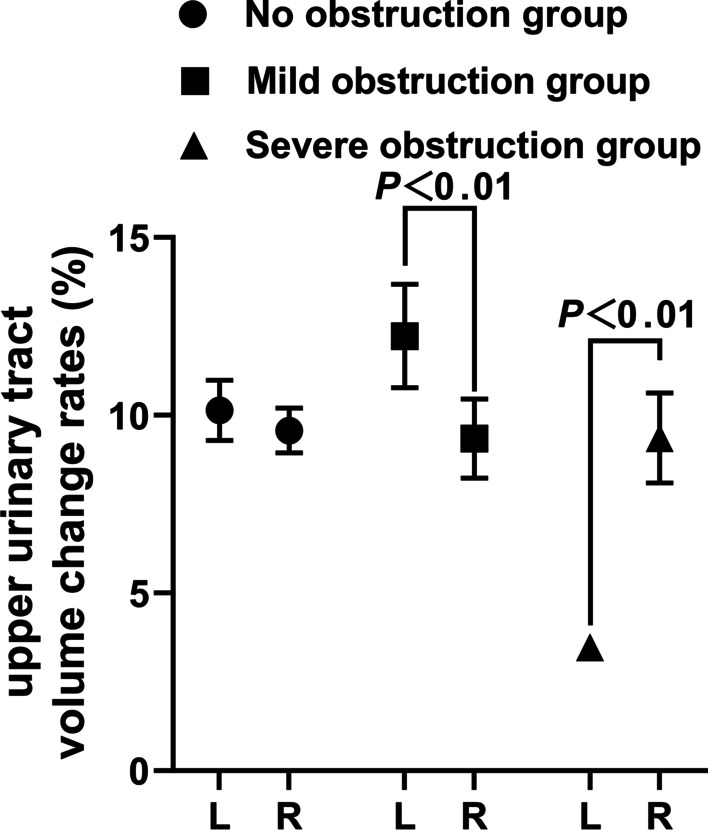
Comparison of upper urinary tract volume change rates among different groups

**Fig. 7:**
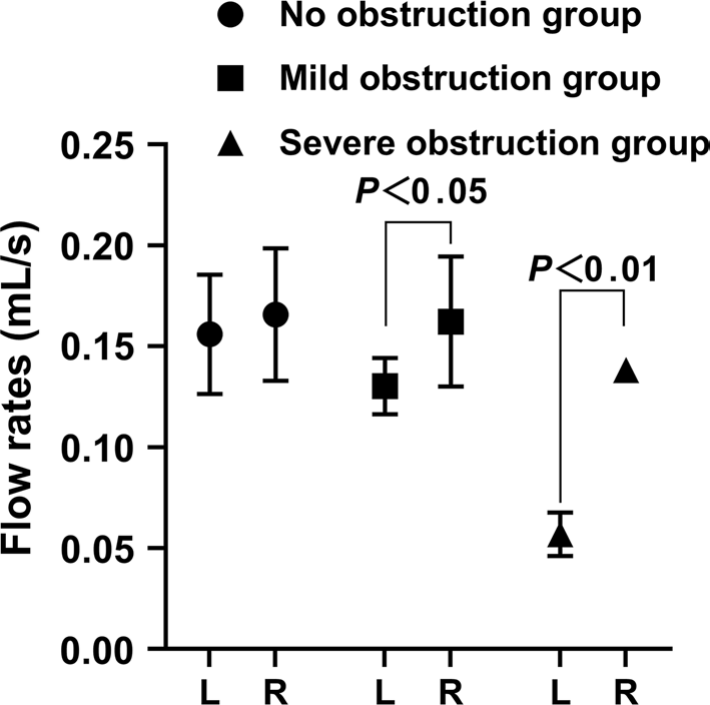
Comparison of calculated flow rates in the upper urinary tract between different groups

**Fig. 8:**
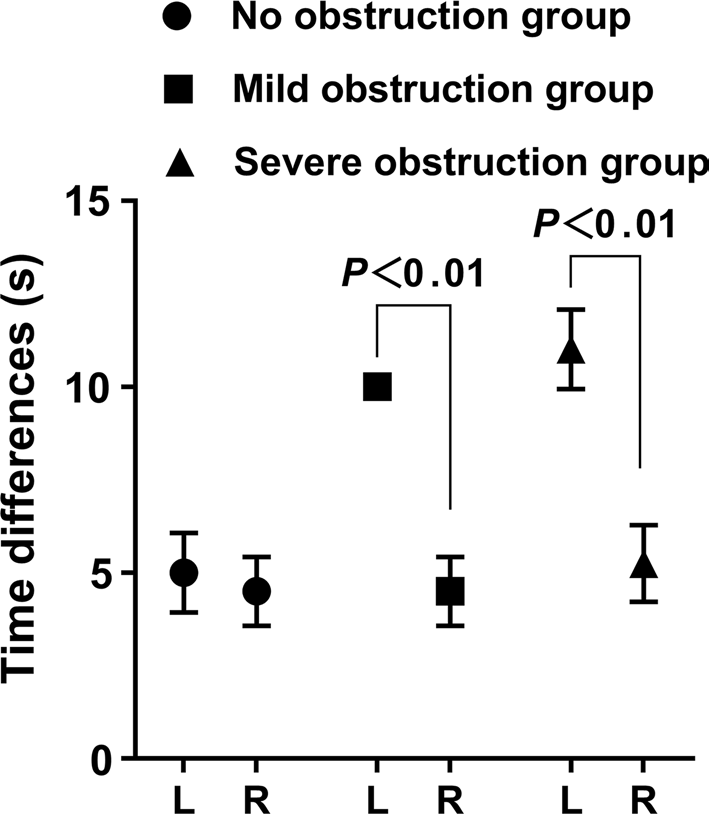
Comparison of time differences between extrema of upper urinary tract volume between different groups

Three groups of different obstructions on the left and right sides of the experimental group and the control group pump function parameters are in line with the normal distribution. Using paired T-test analysis, the statistical results are as follows: Figs. 3, 4, 5, 6, 7 and 8.

## Discussion

### Imaging examination of urodynamics of the upper urinary tract

Urinary tract dilatation does not necessarily involve obstruction, and diagnostic methods that depend on renal function may lead to misdiagnosis [7–9]. The conventional imaging method is static imaging, and functional changes are inferred according to morphological changes in the upper urinary tract [9]. For some unexplained urinary tract dilatations, it is often difficult to judge urodynamics accurately, but this is the focus of urologists. Therefore, previous urodynamic experiments of the upper urinary tract, that is, the renal pelvis constant pressure perfusion test and renal pelvis constant flow perfusion pressure measurement, were used to study and judge the urodynamics of the upper urinary tract. Among them, the renal pelvis CPP test analyses and studies the relationship between the flow rate and pressure of the upper urinary tract [7]. The perfusion fluid pressure was kept constant, and the speed of the liquid passing through the upper urinary tract was measured to determine whether there was upper urinary tract obstruction. If 20 cmH_2_O pressure perfusion was used and the flow rate was less than 10 ml/min, upper urinary tract obstruction was considered. If the flow rate was always less than 4 ml/min, the obstruction was severe, while the flow velocity indicated mild obstruction at 4–10 ml/min [8].

This experimental method was implemented for a quantitative study, but the approach represents an invasive examination for patients with urinary tract dilatation without the need for nephrostomy, which is the main reason to limit the clinical application of this classical technique. Lovasz et al. verified the authenticity and accuracy of the results of upper urinary tract urodynamic diagnosis in nephrostomy patients and tried to improve the experimental method for ascertaining upper urinary tract urodynamics [10], but invasive examination has not been widely used in the clinic. Therefore, in this study, experimental animals were modelled according to the classical CPP test, and multiphase volume data were collected and calculated by 640 layers of DVCT to find a noninvasive quantitative research method to calculate the urodynamics of the upper urinary tract.

### Analysis of urodynamic parameters of the upper urinary tract in this experiment

The perfusion pressure chosen to remain constant in this study was 1.96 kPa (20 cmH_2_O), which was slightly higher than the human renal pelvis pressure of 0.98 kPa (10 cmH_2_O). However, under this perfusion pressure, the urodynamic parameters of the upper urinary tract did not change abnormally, indicating that this pressure was within the safe pressure range of renal pelvic perfusion and was significantly lower than the clinically accepted safe pressure of renal pelvis perfusion during percutaneous nephroscopic surgery (30 mmHg) [11]. Second, because the experimental site is located in Kunming (latitude 25°02: 11\"north, longitude 102°42: 31\"east, average altitude of approximately 1891 m), the background altitude is high and the atmospheric pressure is low, so the standard pressure reference value of 1.96 kPa was adopted.

There is a certain resistance of the left nephrostomy tube itself, and the resistance of the pipe is positively correlated with the length of the tube and inversely correlated with the diameter of the tube. The resistance caused by the thin and long tube is greater. Therefore, if we want to reduce the effect on the perfusion experiment, we should choose pipes with larger diameters and shorter lengths. Before the formal implementation of this experiment, tubes with different diameters were selected for puncture fistulostomy of pig kidneys. It was found that the larger the diameter of the fistulostomy, the more serious the damage to the renal parenchyma, and the larger the diameter of the tube was, the easier it was to cause renal parenchyma rupture. For this reason, combined with the experience of Itoh [12], the catheter of F8 was finally selected to cut its length to the outside body, and a number of side holes were made at the end of the tube at the same time to reduce the resistance of the tube.

The dynamics of the upper urinary tract can be affected by the bladder filling state [13]. In the case of nondiuresis or low urine flow, the filling state of the bladder will not affect the pressure in the renal pelvis, but it will affect the peristaltic frequency of the renal pelvis and ureter and accelerate its frequency, while under high urine flow or in the constant pressure renal pelvis perfusion test, bladder filling will increase the renal pelvis pressure, resulting in a significant slowdown in the speed of urine passing through the upper urinary tract. Therefore, in this experiment, the drainage tube was placed into the bladder through the distal stump of the left ureter to drain the urine in the bladder, thus eliminating the influence of the filling state of the bladder on the experimental results.

The measured urine flow rate of the left upper ureteral stump and the calculated urine flow velocity of the left upper urinary tract decreased with the change in the state of obstruction, and there was no significant difference, indicating that the experimental fistulostomy had no definite effect on the experimental results. The follow-up data measurement are reliable.

### Comparison and analysis of functional parameters of upper urinary tract pumps among groups

The function of the renal pelvis and ureter is to transport urine from the kidney to the ureter and into the bladder for storage until urination. The body works together through a series of mechanisms to achieve this goal. The basic process of regulating ureteral peristalsis is myogenic, initiated by active pacemaker cells located in the renal pelvis, and contractile impulses are transmitted from one ureteral cell to another, making the whole ureter work as a functional complex and pushing the urine into the bladder. The physiology of the urinary tract, particularly the pelvis and ureter, has been studied for nearly 100 years, including its electrical activity and contractile activity. The most important are hydrodynamic factors, such as urine flow rate, which determine the size and pattern of urine boluses, which in turn affect changes in hydrodynamic factors such as peristaltic rhythm, velocity, amplitude and baseline pressure [1]. As early as 1979, Olsen PR determined through animal experiments that renal pelvis pressure, renal pelvis volume and ureteral peristaltic frequency were important characteristics of upper urinary tract urodynamics [14]. Referring to the definition of cardiac pump function, the important characteristics of upper urinary tract urodynamics were designated "upper urinary tract pump function value" in this experiment, including the average volume value of the renal pelvis and upper ureter (ml), volume difference (ml), time difference (s) corresponding to the difference, volume change rate (%) and calculation of urinary flow velocity, which can be used to quantitatively evaluate the urodynamics of the upper urinary tract.

The results showed that the renal pelvis and ureter dilated in the early stage of upper urinary tract obstruction, and the hydronephrosis of the renal pelvis and ureter became more obvious with the deepening of the upper urinary tract obstruction. After left nephrostomy, the average volume of the non-obstruction group, mild obstruction group and severe obstruction group gradually increased, and there were significant differences between the mild and severe groups and the right upper urinary tract. The average volume shows the urine volume of the renal pelvis and upper ureter, which can quantitatively reflect the degree of dilatation of the renal pelvis and upper ureter.

Mudraya et al. [15] found that the intermittent change in the contraction frequency of the renal pelvis is related to the change in the contraction amplitude. The results of this experiment show that when the upper urinary tract is slightly obstructed, the renal pelvis and the upper part of the ureter dilate slightly and stimulate the renal pelvis and ureter to increase their contraction and peristalsis to resist the obstruction so that the contraction frequency of the renal pelvis and ureter smooth muscle is weaker than that of the normal state, but the contraction intensity is increased. Among them, the increase in contraction showed that the difference in the volume positive value was significantly larger than that on the right side, while the volume change rate was slightly higher than that on the right side. The decrease in systolic frequency is shown by the increase in the time difference, but due to the limitation of obstruction, the actual pumped urine volume accumulates in the further dilated urinary tract, which is characterized by a decrease in the urine flow rate. With the further aggravation of urinary tract obstruction, the renal pelvis and upper ureter were further dilated, resulting in overload of smooth muscle tissue, further decrease in contraction frequency and weakening of contraction strength of smooth muscle. The weakening of contraction intensity showed that there was no difference between the positive value difference of volume and that of the right side, while the volume change rate was significantly lower than that of the right side, showing ineffective contraction, resulting in a significant decrease in calculated flow velocity. The decrease in contraction frequency is shown by a further increase in the time difference. The results of this experiment proved the morphological and functional changes of the urinary tract caused by hydronephrosis after obstruction, which was consistent with the results reported by Tillig et al. [16].

In this study, porcine kidneys were scanned by 640-slice DVCT during the excretory phase, and the scanned images were segmented by a Vitreafx workstation to obtain volume images of the renal pelvis and upper ureter. Then, the volume of the upper urinary tract was measured by a workstation, and specific values were obtained; thus, the urodynamics of the upper urinary tract were evaluated accurately. Combining the CPP experiment, one of the classical experiments of upper urinary tract urodynamics, with 640 layers of DVCT, more abundant and practical data describing the renal pelvis and ureteral pump function were obtained than from the CPP experiment, which is an improvement and represents an innovation of the traditional CPP experiment.

However, there are still some limitations in our study. Because this study is a large animal experiment, the experimental tissue is difficult, so the sample size is small; at the same time, the normal state, mild obstruction state and severe obstruction state of the upper urinary tract are applied to the same sample in turn, which may lead to interference between them. Despite this, the examination methods and calculation results of the experiment were applied to patients with urinary calculi according to the approval of the ethics committee of our hospital to obtain satisfactory results.

## Conclusions

In conclusion, our study showed that the continuous dynamic volume scan and measurement of 640-slice DVCT can obtain five pump function datasets of UUT in pigs with acute lower ureteral obstruction. This approach may replace the renal pelvis constant pressure perfusion test as an effective method for noninvasive quantitative imaging evaluation of UUT urodynamics.

## Data Availability

All data generated or analysed during this study are included in this published article.

## Abbreviations

DVCT: Dynamic volume CT
UUT: Upper urinary tract
CPP: Constant pressure perfusion
